# The effect of scientific evidence on conservation practitioners’ management decisions

**DOI:** 10.1111/cobi.12370

**Published:** 2014-08-07

**Authors:** Jessica C Walsh, Lynn V Dicks, William J Sutherland

**Affiliations:** Department of Zoology, University of Cambridge, Downing StreetCambridge, CB2 3E J, United Kingdom

**Keywords:** behavior change, bird predation, conservation synopsis, Delphi method, evidence-based conservation, implementation gap, invasive species, knowledge use, cambio conductual, conservación basada en evidencia, depredación de aves, especies invasoras, falta de datos de implementación, método Delphi, sinopsis de conservación, uso del conocimiento

## Abstract

A major justification of environmental management research is that it helps practitioners, yet previous studies show it is rarely used to inform their decisions. We tested whether conservation practitioners focusing on bird management were willing to use a synopsis of relevant scientific literature to inform their management decisions. This allowed us to examine whether the limited use of scientific information in management is due to a lack of access to the scientific literature or whether it is because practitioners are either not interested or unable to incorporate the research into their decisions. In on-line surveys, we asked 92 conservation managers, predominantly from Australia, New Zealand, and the United Kingdom, to provide opinions on 28 management techniques that could be applied to reduce predation on birds. We asked their opinions before and after giving them a summary of the literature about the interventions’ effectiveness. We scored the overall effectiveness and certainty of evidence for each intervention through an expert elicitation process—the Delphi method. We used the effectiveness scores to assess the practitioners’ level of understanding and awareness of the literature. On average, each survey participant changed their likelihood of using 45.7% of the interventions after reading the synopsis of the evidence. They were more likely to implement effective interventions and avoid ineffective actions, suggesting that their intended future management strategies may be more successful than current practice. More experienced practitioners were less likely to change their management practices than those with less experience, even though they were not more aware of the existing scientific information than less experienced practitioners. The practitioners’ willingness to change their management choices when provided with summarized scientific evidence suggests that improved accessibility to scientific information would benefit conservation management outcomes.

El Efecto de la Evidencia Científica sobre las Decisiones de Manejo de Quienes Practican la Conservación

**Resumen:**

Una justificación mayor de la investigación en el manejo ambiental es que ayuda a quienes lo practican, aunque estudios previos muestran que rara vez se usa para informar sus decisiones. Probamos si quienes practican la conservación enfocada en el manejo de aves estaban dispuestos a usar una sinopsis de literatura científica relevante para informar sus decisiones de manejo. Esto permitió que examináramos si el uso limitado de información científica en el manejo se debe a una falta de acceso a la literatura científica o si se debe a que quienes practican la conservación no están interesados o no son capaces de incorporar la investigación a sus decisiones. En encuestas en línea les preguntamos a 92 practicantes de la conservación, la mayoría de Australia, Nueva Zelanda y el Reino Unido, que nos proporcionaran opiniones sobre 28 técnicas de manejo que podrían aplicarse para reducir la depredación de aves. Les pedimos sus opiniones antes y después de darles un resumen de la literatura sobre la efectividad de las intervenciones. Calificamos la efectividad general y la certidumbre de la evidencia para cada intervención por medio de un proceso de extracción por expertos – el método Delphi. Usamos las calificaciones de la efectividad para evaluar el nivel de entendimiento y de percatación de la literatura de quienes practican la conservación. En promedio, cada participante de la encuesta cambió su probabilidad de usar 45.7% de las intervenciones después de leer la sinopsis de la evidencia. Fue más probable que implementaran intervenciones efectivas y evitar acciones poco efectivas, lo que sugiere que sus estrategias de manejo futuras puedan ser más exitosas que las de práctica actual. Los practicantes con mayor experiencia tuvieron una menor probabilidad de cambiar sus prácticas de manejo que aquellos con menos experiencia, aunque no estuvieron más conscientes de la información científica existente que quienes tenían menos experiencia. La disponibilidad de los practicantes para cambiar sus opciones de manejo al proporcionárseles evidencia científica resumida sugiere que el acceso mejorado a la información científica podría beneficiar los resultados del manejo de la conservación.

## Introduction

Potential application and benefit to society are core reasons for funding and conducting research. Environmental managers obtain their information from a wide range of sources (Cook et al. 2012), though they often rely on past experience and personal opinion rather than the scientific literature to inform their decisions (e.g., Pullin & Knight 2005; Cook et al. 2010; Matzek et al. 2014). Yet there is growing recognition and concern that research is rarely used in management and this has led to a science-practice implementation gap (Knight et al. 2008; Arlettaz et al. 2010; Esler et al. 2010).

Scientific information may be used rarely because the research is insufficiently relevant to management decisions (Fazey et al. 2005; Braunisch et al. 2012; Laurance et al. 2012). Alternatively, practitioners may value scientific evidence (Seavy & Howell 2010; Young & Van Aarde 2011) but have limited access to it (Sunderland et al. 2009; Matzek et al. 2014). Practitioners’ access to scientific information appears to relate directly to the amount of funding, personnel, and resources available (Lauber et al. 2011). Cost is the most important factor in practitioners’ choices of information sources about the management of invasive species (Bayliss et al. 2011). The cost of subscriptions to peer-reviewed journals prevents many practitioners from gaining sufficient access to the scientific literature, particularly in developing countries (Sunderland et al. 2009; Fuller et al. 2014). Even if practitioners have access to journals through their organization or from colleagues at research institutions, the time and skills required to search for, read, and synthesize the primary literature can limit their ability to apply the useful scientific information to their management decisions (Sutherland et al. 2013).

Providing practitioners with a synopsis, or summary, of the literature on specific topics may help to improve access to scientific information. Synopses act as handbooks or databases for busy practitioners because they eliminate the need to directly access and digest the primary literature and enable practitioners to obtain a collated, independent account of the scientific research (Dicks et al. 2014). Synopses are used widely by medical practitioners as a source of validated and synthesized scientific evidence (e.g., *Clinical Evidence* published by the British Medical Journal Group [2014]). We aimed to determine the extent to which improved access to scientific information, presented in a synopsis of the literature, would change management decisions of practitioners and whether the response would vary among types of practitioners.

In experiments providing medical practitioners with easy access to summarized scientific information in different formats, clinical decisions improved, either through confirmation of prior knowledge or a change in practice. When evidence was easily accessible, junior general practitioners corrected 23% of their clinical decisions after conducting literature searches for patient cases and initiated new treatments or diagnostic tests for another 25% of their cases (Sackett & Straus 1998). After general physicians were given relevant evidence from a literature search about specific patients’ treatments, they changed 18% of their decisions (i.e., 1 in 6 patients received a different treatment) (Lucas et al. 2004). When medical practitioners were given access to a hand-held device to obtain rapid, tailored answers to specific clinical questions from a library search, 63% of their subsequent decisions greatly improved (i.e., 20.1% prescribed a more effective treatment, 37.3% learned something new that was applicable to the case, and 5.6% recalled forgotten knowledge relevant to the case) relative to an improvement of 14.9% of decisions in the control group of practitioners who were not sent information from a library search when requested (McGowan et al. 2008). Whether such benefits of increased access to information are true for other fields, such as conservation, appears unknown.

To test this, we used the Bird Conservation Evidence Synopsis (Williams et al. 2013), which collates and summarizes scientific studies that quantify the effect of possible bird conservation interventions. The synopsis is now publicly available as an on-line database (http://www.conservationevidence.com), free electronic document, or printed book.

We focused on 28 possible management interventions from the Bird Conservation Synopsis that aimed to reduce predation of birds by invasive non-native species or problematic native species. Interventions included controlling predators, fencing nesting sites, and altering the predators’ hunting ability or behavior (Williams et al. 2013). We chose the bird predation section of the synopsis because predator control is a particularly relevant topic for bird conservation globally. Predation by invasive or native species has severe and widespread negative impacts on bird populations (Gibbons et al. 2007; Jones et al. 2008). However, choosing options to control predators or prevent predation is challenging, especially when removal of the predator raises conservation or ethical concerns (Redpath et al. 2004; Oppel et al. 2010).

## Methods

### Practitioner Survey

In an initial on-line survey (Supporting Information), we asked conservation practitioners if they had either heard of or used any of the 28 management interventions that address the threat of invasive or problematic species preying on birds of conservation concern (Table1). We also asked the participants how much scientific information they thought existed for each intervention on a scale of 1 (no evidence) to 5 (excellent scientific knowledge). From this question, we calculated their overall awareness-of-evidence score based on their average level of prior knowledge of the scientific information across all interventions relative to the actual existing amount of relevant literature in the Bird Conservation Synopsis (Supporting Information).

**Table 1:** The 28 interventions used to reduce predation on birds and their scores of effectiveness and certainty of evidence as determined by the experts’ scores elicited through the Delphi method

		Certainty of
Intervention	Effectiveness*	evidence
Reduce predation by other species		
(1) remove or control predators to enhance bird populations and communities	65.5	70.5
(2) reduce predation by translocating predators	27.0	20.0
Predator control on islands		
(3) control avian predators on islands	50.0	45.0
(4) control mammalian predators on islands	80.5	77.5
(5) control invasive ants on islands	10.0	15.0
Reduce incidental mortality during predator eradication or control		
(6) distribute poison bait in dispensers to reduce incidental mortality	40.0	25.0
(7) use repellent on baits to reduce incidental mortality	10.0	10.0
(8) use colored baits to reduce incidental mortality	19.5	30.0
Reduce nest predation by excluding predators from nests or nesting areas		
(9) protect bird nesting areas with nonelectric fencing	45.0	48.0
(10) protect bird nesting areas with electric fencing	60.0	59.0
(11) protect nests with individual exclosures or barriers	50.0	50.0
(12) use artificial nests that discourage predation	59.0	54.0
(13) use multiple barriers to protect nests	7.0	17.0
(14) use snakeskin to deter mammalian nest predators	32.5	15.0
(15) use mirrors to deter nest predators	NA	0.0
(16) use naphthalene to deter mammalian predators	0.0	10.0
(17) use ultrasonic devices to deter cats	NA	0.0
(18) protect nests from ants	45.0	16.5
(19) guard nests and prevent predation through direct interference	50.0	30.0
(20) use cat curfews (i.e., require pet cats to be indoors at night) to reduce predation	NA	0.0
(21) use lion dung to deter domestic cats	NA	0.0
(22) play spoken-word radio programs to deter predators	NA	0.0
(23) plant nesting cover to reduce nest predation	27.5	29.5
(24) remove perches used by predators (e.g., trees)	22.0	8.0
Reduce mortality by reducing hunting ability or changing predator behavior		
(25) use collar-mounted devices to reduce predation	47.5	35.0
(26) use supplementary feeding of predators to reduce predation	12.5	20.0
(27) use aversive conditioning to reduce nest predation	9.0	60.0
(28) reduce predation by translocating nest boxes	47.5	25.0

* Abbreviation: NA, no evidence was available about the effect of reducing bird predation.

Once practitioners had completed the initial survey, we sent them a second on-line survey, which they were asked to complete while reading the section on bird predation in the unpublished Bird Conservation Synopsis (Williams et al. 2013). In the second survey (Supporting Information), we asked participants if they were more or less likely to use each intervention after having read the evidence in the synopsis (5-point scale: much less likely, less likely, neither more nor less likely, more likely, much more likely). Practitioners could also indicate *do not know* and *not relevant* if the intervention was not relevant to their conservation problem (e.g., controlling invasive ants on islands is not relevant to a practitioner on the mainland); these cases were excluded from the analysis. The question about the likelihood of changing their management examined whether practitioners found the information in the synopsis useful and whether the evidence provided would influence their future conservation management decisions.

Conservation practitioners who had been involved in the planning or implementation of an intervention to reduce predation on birds within the past 5 years were eligible to complete the survey. We used opportunistic snowball sampling and aimed to obtain a sample of eligible practitioners from different types of organization and with varying levels of experience from a wide range of countries. The limitations of snowball sampling prevented us from estimating how representative our sample was of the targeted population of practitioners working to reduce bird predation. We could not accurately determine the response rate because we were unable to track how many eligible people received the survey. Hence, we were cautious about making generalized statements about the wider conservation practitioner community from the results of this study.

### Expert Elicitation Process

The Delphi method is an expert elicitation process that entails asking a panel of experts to provide an estimate, score, or opinion for a question with an unknown or uncertain answer and then to revise and update their own estimate after seeing the results of other anonymous panel members in subsequent rounds of scoring (Rowe & Wright 2011). We used the Delphi method to quantify the effectiveness and certainty of evidence of each intervention used to reduce bird predation in 3 rounds of on-line scoring.

In the first round of scoring, we asked experts to give each intervention a score of its effectiveness at reducing predation of birds (from −10 if detrimental to +100 if highly effective), based on the evidence in the Bird Conservation Synopsis, and a score of the certainty of the evidence on its effectiveness (0 to +100), which was determined by the quantity and quality of the evidence in the synopsis. We also asked the experts to provide a justification for their scores. They were given instructions on how to score the interventions and an explanation of the Delphi process (Supporting Information).

In both the second and third rounds of scoring, panel members were given the anonymous scores of effectiveness and certainty of evidence as well as comments of other experts from the previous round and were asked to reassess their own scores, once again providing a reason for changing or keeping their score. The median of the experts’ final scores from the third round formed each intervention's overall measure of effectiveness and certainty of evidence. These data were used as explanatory variables to test if practitioners interpreted the evidence correctly and whether providing the evidence improved management decisions.

We followed recommendations from recent reviews and critiques of the Delphi method to ensure the process was rigorous and achieved the most accurate estimates for each variable (Supporting Information). We invited people to be on the expert panel who had extensive experience in either bird predation research or management, based on their publication record or personal contacts. Ten of the 12 invited experts participated in the Delphi process and completed all 3 rounds (except for one expert who missed the second round of scoring). Details about the reduction in variation of scores between rounds are provided in Supporting Information. The 5 interventions for which no evidence was available were automatically given a score of unknown effectiveness, a certainty of evidence score of 0, and were not included in the Delphi process.

### Practitioners’ Likelihood of Using the Evidence

To determine if practitioners were interpreting the evidence correctly, we fitted 2 log-linear models between the direction of a practitioner's likelihood of using an intervention in the future (i.e., more likely, no change, less likely, or do not know) and the interventions’ effectiveness and certainty of evidence. For this analysis, we converted the effectiveness and certainty of evidence scores into categorical variables with the quartiles as cut-off points for each category.

To identify the types of practitioners who were more willing to use the evidence provided in the synopsis to inform their management decisions, we modeled the practitioners’ likelihood of changing their opinion about a management intervention after reading the evidence (change or no change) with a generalized linear mixed model with a binomial error distribution and logit link function. The full model included the practitioners’ past exposure to the interventions (not heard of, heard of, and used), their prior knowledge of the scientific literature for each intervention, the number of years experience they have in the conservation field, their role as an advisor or manager, the type of organization they work for (government agency, nongovernmental organization, and other), the region where they work (Australia, New Zealand, United Kingdom, and other countries), and the effectiveness and certainty of evidence of interventions. The full model also included 5 relevant 2-way interactions between 4 of the explanatory variables: practitioners’ prior knowledge of literature about an intervention, their past exposure to an intervention, their experience, and the effectiveness of interventions. Practitioner and intervention were included as random effects. We tested continuous variables for normality with diagnostic plots prior to modeling, though no transformations were required. We excluded interventions that had no evidence available or that participants had selected as *not relevant* in the second survey, leaving 1050 data points from 92 practitioners and 23 interventions. Models with an Akaike information criterion (AIC) value within 2 from the model with the lowest AIC value were considered the best-fitting models (Burnham & Anderson 2002). We used the lmer function in the lme4 package from the program R, version 2.15.1 (R Development Core Team 2005).

## Results

We received 112 complete and 4 partial responses from eligible practitioners for the initial survey and 90 completed and 2 partial responses for the second survey (80.5% response rate for the second survey). We included data from partial responses in the analyses where possible. Conservation practitioners from 26 countries, mainly the United Kingdom, New Zealand, and Australia, and from a diverse range of organizations participated in the study (Table2). The participants’ experience in the conservation field ranged from 1 to over 45 years (mean 18.5 years).

**Table 2:** Demographic characteristics of conservation practitioners (*n* = 92) surveyed across type of organization, role as a conservation practitioner, and region of work

		Number of	
Variable		respondents	%
Organization	national government organization	24	0.26
	state government organization	10	0.11
	local government organization	0	0.00
	nongovernmental organization—international	4	0.04
	nongovernmental organization—national	27	0.29
	nongovernmental organization—local	11	0.12
	university or research institution	9	0.10
	business or consulting firm	6	0.07
	individual	1	0.01
Role	managing	47	0.51
	advising	30	0.33
	both	4	0.04
	other	11	0.12
Region*	New Zealand	22	0.24
	Australia	21	0.23
	United Kingdom	20	0.22
	United States	8	0.09
	Canada	3	0.03
	other – Asia and Pacific	9	0.10
	other – Europe	4	0.04
	other – Africa	3	0.03
	other – South America and Caribbean	2	0.02

* Countries with 1 or 2 participants were combined into larger geographical regions.

On average, practitioners had heard of 57.1% of the possible 28 interventions used to reduce predation of birds by invasive or problematic species (min = 14.3%, max = 100.0%) and had previously implemented 18.5% of the listed interventions (min = 3.6%, max = 64.3%). There was a positive significant relationship between the effectiveness of an intervention and the proportion of practitioners who had used the intervention (quasibinomial logistic regression, β = 0.035, SE 0.009, *t* = 3.647, *P* = 0.002, df 21). A similar relationship was found between the certainty of evidence for each intervention and the proportion of practitioners who had used it (quasibinomial logistic regression, β = 0.040, SE 0.008, *t* = 5.239, *P* < 0.001, df 26).

On average, practitioners changed their opinions on 45.7% of the listed interventions after reading the evidence summarized in the Bird Conservation Synopsis. That is, they were willing to use the summary of the literature to override their previous judgments in light of the newly available evidence for almost half of the interventions. Over 90% of all practitioners indicated they would change their use of at least one intervention (85 of 92 participants). The majority of the other practitioners who were not influenced by the evidence were already using, on average, 79% of the effective interventions (i.e., those with an effectiveness score in the upper quartile; effectiveness >48) and had no reason to change their existing practices. Of the practitioners who were more likely or much more likely to use an intervention, over three-quarters on average had not previously used that intervention, indicating these were true changes of intent, rather than strengthening of support for their existing management practices.

**Figure 1:**
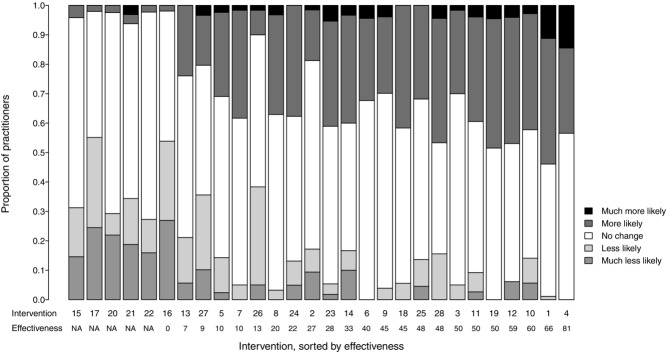
Proportion of practitioners more likely, less likely, or neither more nor less likely to use an intervention after reading the Bird Conservation Synopsis, ordered by ascending effectiveness of the intervention. Codes for interventions are defined in Table1.

After reading the evidence, respondents said they would be more likely to use those interventions that were effective at reducing bird predation and less likely to use interventions that were ineffective or for which there was no evidence of their effectiveness (Fig.1: log-linear model with Poisson distribution, residual deviance = 253.8, *p* < 0.001, df 12). There was a similar association between an intervention's certainty of evidence and the likelihood of a practitioner using it after reading the evidence (log-linear model, residual deviance = 178.8, *P* < 0.001, df 9). Effectiveness and certainty of evidence for interventions were significantly correlated (Spearman's rank correlation coefficient, ρ = 0.829, *P* < 5.15×10^−8^, *n* = 23). These results show that the practitioners were able to accurately interpret the content and quality of the evidence provided in the synopsis.

**Figure 2:**
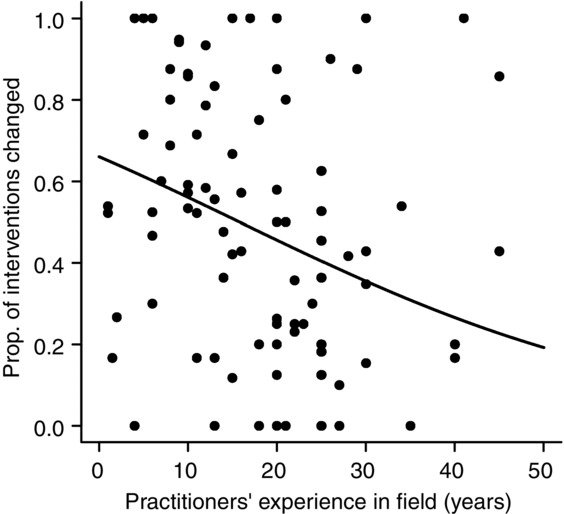
Proportion of management interventions that each practitioner changed their likelihood of using relative to the practitioner's level of experience in the conservation field (plotted using parameter estimates from model 1 [Table3]).

Practitioners with more experience in the conservation field were significantly less likely to change their mind about interventions (Fig.2, Table3: models 1 & 2). The practitioners’ past exposure to interventions was also in one of the 2 best-fitting models explaining the likelihood of practitioners changing their management actions after reading the evidence (Table3: model 2). This model showed a significant interaction between practitioner's experience and their past exposure to the intervention; the trend between experience and likelihood of changing was the opposite for practitioners who had previously not heard of or used the intervention. Practitioners with more experience who had not heard of an intervention were much more likely to change their management than practitioners with less experience who were unfamiliar with the intervention. We suspect this is an artifact of the low frequency of records in this category, given that more experienced practitioners had already heard of most interventions and that most of these records were excluded from the analysis because of missing data about their prior knowledge of the existing scientific information.

**Table 3:** Ten best-fitting models showing which practitioners were more likely to change their mind about an intervention after reading the evidence presented in the Bird Conservation Evidence Synopsis.*a*

Model	Intercept	Prior	Past exposure to		Effective-	Certainty							Past exposure^*^			
		knowledge	intervention		ness	of evidence	Experience	Organization		Region			experience		AIC	Δ AIC
			heard of	used				NGO	other	NZ	UK	other	heard of	used		
1*b*	0.661						−0.042								1272.0	0.0
2*b*	−0.313		1.210	0.934			0.030						−0.086	−0.071	1272.4	0.4
3	0.251									−0.475	−1.114	0.047			1274.7	2.7
4 (null)	−0.099														1275.5	3.5
5	0.631		0.043	0.029			−0.042								1276.0	4.0
6	−0.049	−0.317													1276.4	4.4
7	−0.032				−0.002										1277.3	5.3
8	−0.158					0.002									1277.4	5.4
9	−0.069							−0.098	0.068						1279.3	7.3
10	−0.109		0.028	−0.007											1279.4	7.4

a The default categories in the model output are as follows: past exposure to an intervention—neither heard of or used; organization type—government organization; region—Australia. The average variances for the random effect variables intervention and practitioner across the 10 models are 0.14 and 2.18, respectively.

b Selected as the best models based on their low AIC values.

**Figure 3:**
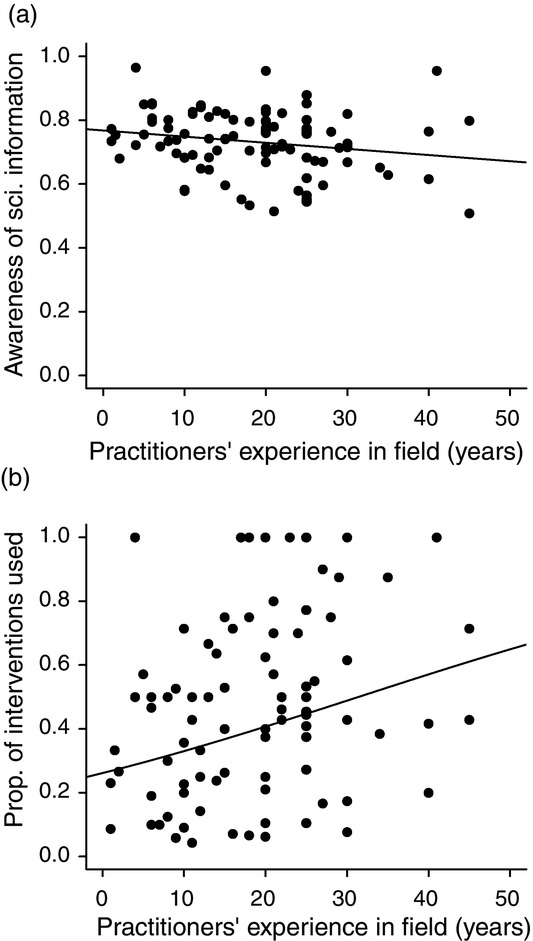
The relationship between practitioners’ number of years of experience in the conservation field and (a) their average awareness of the scientific information prior to reading the evidence (1.0, perfect knowledge of the extent of research for all intervent-ions; 0.0, no knowledge of the extent of research for any intervention) (linear regression, β = −0.002, SE 0.001, t = −1.683, P = 0.096, df 90) and (b) the propo-rtion of interventions they used prior to reading the synopsis (quasibinomial logistic regression, β = 0.033, SE 0.011, t = −2.966, p = 0.004, df 90).

To understand the importance of practitioners’ experience in their likelihood of changing their management strategies, we investigated this relationship further. Either practitioners with more experience acquired a large knowledge base through past reading, practice, or intuition so that the content in the evidence we provided was not new or the practitioners did not take account of what the evidence suggested, even if it contradicted their existing knowledge and past management practices. We tested each of these hypotheses by assessing the relationship between the practitioners’ experience and their level of awareness of the evidence prior to the study. Practitioners with more years of experience in the conservation field were not more aware of the existing scientific information prior to reading the synopsis of literature; in fact, the relationship was negative, but not significantly so (Fig.3a). More experienced practitioners had also used a greater proportion of interventions than less experienced practitioners before reading the synopsis (Fig.3b).

## Discussion

### Importance of Access to Scientific Information

One reason for conservation science to be funded and conducted is so that it can be used by practitioners. Though in practice, practitioners are unable to access most of the literature because the majority of studies are published in journals requiring subscriptions (Pullin & Knight 2005; Matzek et al. 2014). The evidence provided in the Bird Conservation Synopsis is not new because all the summarized studies were published elsewhere and in theory should have been accessible to practitioners beforehand. However, our results show that by improving respondents’ access to scientific evidence by providing them with this information in an easily accessible, clearly summarized format, they are likely to use it to inform and change almost half of their environmental management decisions. This finding is important because it suggests that practitioners find evidence about the effectiveness of interventions useful and relevant, that most are willing to use it, and that access to this information is one of the main barriers to research utilization in conservation.

We also showed that Conservation Evidence Synopses are an effective way to provide conservation practitioners with a free, easily accessible source of scientific information and could help to overcome the difficulties of finding the best available evidence in time- and resource-limited situations. The practitioners’ willingness to change indicates the Bird Synopsis and the underlying research provided them with relevant, useful information to support their decisions. The synopsis of evidence may also have confirmed practitioners’ prior knowledge, contributed wider general knowledge valuable for future decisions, or given support for current management practices. These are all possible indicators of evidence use we did not capture in this study.

### Evidence for an Evidence-Based Conservation Approach

Our results show the value of improving practitioners’ access to synopses of scientific research and the benefits of using evidence in their decision making. Many practitioners in the study who were more likely to use effective interventions had not previously used them, demonstrating the potential for genuine changes in conservation management as a result of easily accessible summaries of evidence. For example, by simply providing summaries of the studies that tested the effectiveness of using artificial nests that discourage predation (intervention 12), 47% of all practitioners in this study said they would be more likely to use them in the future; 78% of these had not previously used them. With limited access to summarized scientific information, practitioners may remain unaware of the most suitable and effective management options and be limited in their capacity to make effective decisions.

### Influence of Evidence on Practitioners

Despite practitioners expressing a desire to change on average 46% of their management actions when given the existing evidence in a useable format, in the majority of occasions the participants stayed with their current management. The most likely explanation for this is that their existing means of collating information and deciding upon effective solutions was sufficient. The major reason for survey participants not to change their management preferences was that they were already using the effective interventions. For example, 69% of the practitioners who did not change their likelihood of using the most effective management intervention (i.e., controlling mammalian predators on islands, intervention 4: Table1) had previously implemented it. This is also supported by the significant positive relationships between the proportion of practitioners who had previously used an intervention and its effectiveness and certainty of evidence. Thus, although we found that providing evidence improved decision making, we also found that existing approaches, such as use of experience and expert opinion, do partly work.

Participants with more experience were less likely to change their practices and continue with their current management strategies (Fig.2). They had similar (or perhaps even lower) levels of awareness of the existing scientific evidence relative to less experienced practitioners (Fig.3a), which suggests they had not read the literature, and may have successfully identified effective management techniques through experience (e.g., trial and error) (Fig.3b) and advice from colleagues. However, the effectiveness and efficiency of management actions may be reduced if more experienced practitioners tend to rely on outdated information and are reluctant to take on new evidence. In contrast, Young and Van Aarde (2011) found that South African practitioners with intermediate levels of experience (7–20 years experience) working on elephant management are less likely to use scientific information for applied management decisions than practitioners with more experience who are more likely to incorporate science into their decisions. This finding mirrors a trend observed in the uptake of evidence-based practice in the medical field (Smith & Rennie 2014).

A new generation of practitioners with scientific literacy skills and possibly a deeper appreciation for the use of scientific research in management may resolve this research utilization gap, as a result of the recent emphasis on graduate education and training for conservation professionals (Duchelle et al. 2009; Courter 2012). Multifaceted training courses in the medical field on evidence-based practices increase the use of scientific information in practice and improve treatment outcomes (Straus et al. 2005); thus, professional development and continued education for practitioners may be one way to reduce stagnant information flow in conservation (Shanley & Lopez 2009; Cook et al. 2013).

Practitioners who participated in this study may have been unwilling to change when the evidence for an intervention was sparse, conflicting, or not applicable to their specific context. They may not have changed their opinions about interventions because of other factors, such as an intervention's practicality, cost, or political, institutional, and cultural values that need to be considered alongside the scientific research when making decisions. For these reasons, better access to scientific evidence is important to improve management decisions, but alone it may not be sufficient to increase its use in practice, as demonstrated in the medical literature (Baker et al. 2010; Zwolsman et al. 2012).

### Defining the Use of Evidence and Other Caveats

Our results should be considered in light of several limitations. We measured people's intentions of changing their behavior as a result of access to summaries of scientific evidence, rather than observing actual changes. Attitudes and intentions may not always reflect behavior (Ajzen 2005), and the participants’ initial responses to the evidence may not lead to changed management actions. However, willingness to change is an important step in knowledge uptake; thus, it is a valid indicator for potential behavior change (Nutley et al. 2007).

The majority of the survey participants came from English speaking developed countries, where most of the research on evidence-based conservation has been conducted (e.g., Pullin & Knight 2005; Cook et al. 2012; Ewen et al. 2013). We do not know if these results reflect practitioners’ willingness to use synopses of scientific information from less developed countries, though there are several factors that could influence their uptake of the evidence. Conservation managers in these countries tend to have poorer access to the scientific literature than those in developed nations (Sunderland et al. 2009; Gossa et al. 2014). The inherent bias in the published conservation literature and in data toward northern hemisphere regions and temperate conservation issues should also be accounted for because this information may have little relevance to the problems faced in the “global south” (Karlsson et al. 2007; Amano & Sutherland 2013). However, given the fewer resources available and lower scientific literacy rates in less developed countries relative to the practitioners sampled in this study (Sunderland et al. 2009), it is possible that these practitioners may be even more willing to use the evidence provided in free summaries of literature rather than the primary literature. Further research focusing on conservation practitioners in these regions is needed to better understand their information needs and current use of scientific information (but see Gossa et al. 2014).

We were unable to remove potential biases in the types of practitioners who responded to our surveys due to the snowball-sampling design and because we relied on a small sample of self-selected participants. The survey may present an overestimate of the proportion of practitioners who would change their management strategies because practitioners who are skeptical of science and reluctant to use research would be less likely to complete the survey. As with any on-line survey, ours was restricted to people with good access to the Internet and time available to participate. The Hawthorne effect (i.e., participant responses are influenced by the knowledge that they are being studied) was unavoidable. Because of this, participants may have indicated a change in their likelihood of using interventions only to satisfy or impress the researchers.

### Improving Access to Scientific Information

Our results suggest it is worthwhile to improve access to scientific information for conservation practitioners. This access could be improved through open access publication (Fuller et al. 2014), greater financial and organizational support for science communication and knowledge exchange programs, and the collation and synthesis of scientific evidence.

Summarizing and evaluating research findings in clear, concise, relevant, and freely accessible packages overcome the problems of physical availability of the literature and reduces the time and skills practitioners need to digest the information. Several initiatives exist for this purpose, including the Conservation Evidence Synopses and systematic reviews (Collaboration for Environmental Evidence, http://www.environmentalevidence.org). These systematic methods of collating scientific evidence have successfully revolutionized clinical medical practice (Sackett et al. 1996), and similar efforts in the environmental sector may also be equally influential.

## Supporting Information

A description of the Bird Conservation Synopsis (Appendix S1), the questions from the on-line surveys (Appendix S2), additional details of methods (Appendix S3), and instructions given to the expert panel (Appendix S4) are available on-line. The authors are solely responsible for the content and functionality of this material. Queries (other than absence of the material) should be directed to the corresponding author.
