# Supplementary material for: The effect of scientific evidence on conservation practitioners’ management decisions
Source: Conserv Biol. 2014 Aug 7;29(1):88–98. doi: 10.1111/cobi.12370 (PMC4515094; doi:10.1111/cobi.12370)
Supplement: Supplementary file 1 [file cobi0029-0088-sd1.pdf]

**Appendix S1: Bird Conservation Evidence Synopsis**

*The effect of scientific evidence on conservation practitioners' management decisions*

Authors: Jessica C. Walsh*, Lynn V. Dicks and William J. Sutherland
* j.walsh@zoo.cam.ac.uk

The Bird Conservation Synopsis belongs to a series of synopses that are designed to collate and summarize the results of existing scientific studies (published in peer reviewed journals and the grey literature) to make the vast and sometimes inaccessible conservation science literature available to users. Only studies that test the effectiveness of a conservation management action are included in the synopses.

The findings and other important details from each relevant study are summarized into a paragraph, which are then in turn compiled into 'key messages' for each action or 'intervention'. The interventions are separated into chapters, categorised by their associated threat. An international expert advisory board endorses the list of interventions and the criteria for the literature search and approves the final content of the key messages and summaries of each study. The synopses do not explicitly state which interventions are effective, nor do they give advice on how to implement each intervention. This is because the relevance of the original scientific studies needs to be assessed in the context of each conservation management decision.

A section of the Bird Conservation Synopsis was used for this study. There was large variation in the quantity of evidence available for each intervention to reduce bird predation, ranging from 0 to 35 studies per intervention of varying quality (mean 4.6 studies per intervention). For an extract from the synopsis about the effectiveness of controlling avian predators on islands (intervention 3: Table 1) visit the Conservation Evidence to find the database (www.ConservationEvidence.com/actions/372) or the complete synopsis, which is freely downloadable from this website (pages 249-250 for this intervention). It shows the key messages collated from 10 studies and the summarised paragraphs for each of the studies.
