# Supplementary material for: The effect of scientific evidence on conservation practitioners’ management decisions
Source: Conserv Biol. 2014 Aug 7;29(1):88–98. doi: 10.1111/cobi.12370 (PMC4515094; doi:10.1111/cobi.12370)
Supplement: Supplementary file 2 [file cobi0029-0088-sd2.pdf]

Appendix S2: Surveys sent to practitioners before and after reading the evidence

The effect of scientific evidence on conservation practitioners' management decisions

Jessica C. Walsh*, Lynn V. Dicks and William J. Sutherland
* j.walsh@zoo.cam.ac.uk

Survey 1: Evidence on interventions to reduce bird predation

Consent Form

Evidence on the effectiveness of bird conservation interventions that reduce their predation

This survey is part of a research project led by the University of Cambridge and funded by a UK Commonwealth Scholarship.

We expect the survey to take you about 20 minutes. As an incentive for your efforts, we will provide you with a summary of the scientific evidence for each intervention which reduces bird predation, taken from the 'Bird Conservation' synopsis.

Before you start, please look at the Project Information Sheet.

Once you accept to participate in the survey, you will be able to move back and forth through the survey, up until the last page where it will ask you to submit your answers. You should also be able to save your answers and complete the survey at a later stage.

Please read the following statements:

- I have read and understood the Project Information Sheet dated 28 October 2011.
- I understand that my participation is voluntary and I am free to withdraw at any time.
- All the information I provide will be stored (and backed up) on secure computers, with access only by the immediate research team.
- No personal details such as my name or email address will be revealed to people outside the project.
- The project results will be presented at conferences and written up in scientific journal papers with scores aggregated and no means of linking them to individual people or organisations.
- No video or audiotaping will take place as part of this study.

I agree to participate in this research survey*

( ) Yes

Your details

Please fill in your details.

Name: ____

Name of organisation: ____

Country: ____

Email address: ____

Number of years working in the conservation field:
____

Which of these are you primarily responsible for?

( ) Managing one or several reserves, e.g. warden or park ranger

( ) Advising wardens or park rangers on the management of one or several reserves

( ) Other: ____

Knowledge and use of interventions

The remaining sections of the survey will ask you three questions about 28 different interventions that could be used to manage bird populations or communities from predation by invasive or problematic species.

There will be space at the end of the survey to comment on any intervention if you wish.

Reduce predation by other species

Please answer the three questions below for this management intervention.

Predator control or eradication could be achieved through various strategies, including baiting, trapping or hunting.

	Have you heard of this intervention?	Have you ever used it or been involved in implementing it?	How much scientific evidence do you think there is about whether this intervention benefits bird species?				
	Yes	Yes	1 No evidence	2	3	4	5 Excellent scientific understanding
Remove or control predators to enhance bird populations and communities	[ ]	[ ]	( )	( )	( )	( )	( )

Reduce bird mortality by reducing hunting ability or changing predator behaviour

Please answer the three questions below for all management interventions.

Note:

Providing supplementary food for the predator may reduce predation pressure on the threatened bird species.

Collar-mounted devices, such as bells, can be fitted to collars of predators, which alerts birds before being caught.

Aversive conditioning is when predators are taught to associate bird nests and eggs with unpleasant smells or tastes, which may reduce the risk of predation.

Translocating nest boxes may reduce predation, as many predators optimise their hunting by searching areas where they have previously been successful.

	**Have you heard of this intervention?**	**Have you ever used it or been involved in implementing it?**	**How much scientific evidence do you think there is about whether this intervention benefits bird species?**				
	**Yes**	**Yes**	**1 No evidence**	**2**	**3**	**4**	**5 Excellent scientific understanding**
Use supplementary feeding of predators to reduce predation	[ ]	[ ]	( )	( )	( )	( )	( )
Use collar-mounted devices to reduce predation	[ ]	[ ]	( )	( )	( )	( )	( )
Use aversive conditioning to reduce nest predation	[ ]	[ ]	( )	( )	( )	( )	( )
Reduce predation by translocating nest boxes	[ ]	[ ]	( )	( )	( )	( )	( )

Reduce nest predation by excluding predators from nests or nesting areas

Please answer the three questions below for all management interventions.

	Have you heard of this intervention?	Have you ever used it or been involved in implementing it?	How much scientific evidence do you think there is about whether this intervention benefits bird species?				
	Yes	Yes	1 No evidence	2	3	4	5 Excellent scientific understanding
Use artificial nests that discourage predation	[ ]	[ ]	( )	( )	( )	( )	( )
Guard nests and prevent predation through direct interference	[ ]	[ ]	( )	( )	( )	( )	( )
Use mirrors to deter nest predators	[ ]	[ ]	( )	( )	( )	( )	( )
Use 'cat curfews' to reduce predation	[ ]	[ ]	( )	( )	( )	( )	( )
Play spoken-word radio programmes to deter predators	[ ]	[ ]	( )	( )	( )	( )	( )
Use lion dung to deter domestic cats	[ ]	[ ]	( )	( )	( )	( )	( )
Use snakeskin to deter mammalian nest predators	[ ]	[ ]	( )	( )	( )	( )	( )
Use naphthalene to deter mammalian predators	[ ]	[ ]	( )	( )	( )	( )	( )
Use ultrasonic devices to deter cats	[ ]	[ ]	( )	( )	( )	( )	( )

Plant nesting cover to reduce nest predation	[ ]	[ ]	( )	( )	( )	( )	( )
Remove perches used by predators, e.g. trees	[ ]	[ ]	( )	( )	( )	( )	( )
Protect nests from ants	[ ]	[ ]	( )	( )	( )	( )	( )
Protect bird nesting areas using electric fencing	[ ]	[ ]	( )	( )	( )	( )	( )
Protect bird nesting areas using non-electric fencing	[ ]	[ ]	( )	( )	( )	( )	( )
Protect nests with individual exclosures/barrier	[ ]	[ ]	( )	( )	( )	( )	( )
Use multiple barriers to protect nests, e.g. a fence and individual nest exclosures	[ ]	[ ]	( )	( )	( )	( )	( )

Reduce incidental mortality during predator control

Eradication and control programmes run the risk of damaging the bird populations they are meant to protect, as birds may take poison or get caught in traps. The following interventions may reduce accidental mortality of non-target species, without compromising the effectiveness of target species control.

Please answer the three questions below for all management interventions.

	Have you heard of this intervention?	Have you ever used it or been involved in implementing it?	How much scientific evidence do you think there is about whether this intervention benefits bird species?				
	Yes	Yes	1 No evidence	2	3	4	5 Excellent scientific understanding
Use repellent on baits to reduce incidental mortality	[ ]	[ ]	( )	( )	( )	( )	( )
Distribute poison bait using dispensers to reduce incidental mortality	[ ]	[ ]	( )	( )	( )	( )	( )
Reduce predation by translocating predators	[ ]	[ ]	( )	( )	( )	( )	( )
Use coloured baits to reduce incidental mortality	[ ]	[ ]	( )	( )	( )	( )	( )

Predator control on islands

Please answer the three questions below for all management interventions.

	Have you heard of this intervention?	Have you ever used it or been involved in implementing it?	How much scientific evidence do you think there is about whether this intervention benefits bird species?				
	Yes	Yes	1 No evidence	2	3	4	5 Excellent scientific understanding
Control mammalian predators on islands	[ ]	[ ]	( )	( )	( )	( )	( )
Control invasive ants on islands	[ ]	[ ]	( )	( )	( )	( )	( )
Control avian predators on islands	[ ]	[ ]	( )	( )	( )	( )	( )

Comments

Do you have any comments about any of the interventions? Please feel free to tell us about other interventions we have not included in this survey.

____

____

Thank You!

Thank you for completing our survey. Your response is very important to us. We will soon send you a summary on the evidence for each bird predation intervention. In the mean time, please have a look at our website www.conservationevidence.com. We look forward to collating your responses in the second survey.

Survey 2: The evidence on interventions to reduce bird predation

---

Evidence on interventions to reduce bird predation

Welcome to Part 2!

Thank you for returning to complete the second part of our study. Please finish and submit this final survey to ensure that your response can be included in the data analysis.

For more details, please read the Project Information Sheet.

The survey will take 10 minutes to complete. Though, please allow yourself extra time to read the information in the Bird Conservation Synopsis (Williams *et al*., in prep) about the interventions that are relevant to you.

You are able to move forwards and backwards through the survey until the final page which asks you to submit your answers. You are able to save your answers and continue the survey later if required.

If a 'time out' error occurs in the survey, please press 'Refresh' and continue from the page that you were on - your previous answers should already be recorded.

Williams, D.R., Pople, R.G., Showler, D.A., Dicks, L.V., Child, M.F., zu Ermgassen, E.K.H.J. and Sutherland, W.J. in prep. Bird conservation: evidence for the effects of interventions. Synopses of Conservation Evidence Series, Vol. 2. Pelagic Publishing, Cambridge.

Bird Predation Survey Instructions

Please answer the survey in the context of your current situation.

If an intervention could potentially be beneficial to your specific problem(s) on bird predation, we ask that you read the corresponding information in the synopsis before answering the question for that intervention.

If you think an intervention is irrelevant to your specific project or work, please select the 'Not Applicable (NA)' option. You do not need to read the information for interventions that are irrelevant to you.

The summaries on the effectiveness of each intervention include brief key messages, followed by background information, a detailed summary of each relevant study and then the bibliography.

The summaries can be found by clicking on the links throughout the survey. If you would prefer to print out the information (attached in the email), the page numbers correspond to the location of the intervention in the synopsis.

Your details

Before you begin, could you please fill in your details.*

These are needed to link participants' responses with their answers from the initial survey. Any contact details that could be used to identify individuals will be removed before the data analysis and kept confidential.

*We require at least one of these details.

Name: ____

Email address: ____

Reduce predation by other species

One approach to reduce predation is to eradicate or control predator populations, through poison baiting, trapping or hunting. However, in some situations, translocating the predator population may be possible or preferred.

Having read the information on the effectiveness of these interventions provided in the bird synopsis, are you more or less likely to use each intervention in the future?

	**Much more likely**	**More likely**	**No change**	**Less likely**	**Much less likely**	**Don't know**	**NA**
Remove or control predators to enhance bird populations and communities Pg. 2	( )	( )	( )	( )	( )	( )	( )
Reduce predation by translocating predators Pg. 13	( )	( )	( )	( )	( )	( )	( )

Predator control on islands

Having read the information on the effectiveness of these interventions provided in the bird synopsis, are you more or less likely to use each intervention in the future?

	Much more likely	More likely	No change	Less likely	Much less likely	Don't know	NA
Control avian predators on islands Pg. 14	( )	( )	( )	( )	( )	( )	( )
Control mammalian predators on islands Pg. 17	( )	( )	( )	( )	( )	( )	( )
Control invasive ants on islands Pg. 28	( )	( )	( )	( )	( )	( )	( )

Reduce incidental mortality during predator eradication or control

Eradication and control programmes run the risk of damaging the bird populations they are meant to protect, as birds may take poison or get caught in traps. The following interventions may reduce accidental mortality of non-target species, without compromising the effectiveness of target species control.

A study that looked at whether birds take bait unintentionally during predator control is summarised on Pg. 28.

Having read the information on the effectiveness of these interventions provided in the bird synopsis, are you more or less likely to use each intervention in the future?

	Much more likely	More likely	No change	Less likely	Much less likely	Don't know	NA
Use coloured baits to reduce incidental mortality Pg. 31	( )	( )	( )	( )	( )	( )	( )
Use repellent on baits to reduce incidental mortality Pg. 30	( )	( )	( )	( )	( )	( )	( )
Distribute poison bait using dispensers to reduce incidental mortality Pg. 29	( )	( )	( )	( )	( )	( )	( )

Reduce predation by excluding predators from nests and other areas

Predators can have a devastating impact on bird populations through predating eggs and chicks too young to defend themselves or run away.

One systematic review has compared the effectiveness of various methods of excluding predators from nesting sites and possible adverse effects. The summary of the findings is on Pg. 32.

Having read the information on the effectiveness of these interventions provided in the bird synopsis, are you more or less likely to use each intervention in the future?

	**Much more likely**	**More likely**	**No change**	**Less likely**	**Much less likely**	**Don't know**	**NA**
Protect bird nesting areas using non-electric fencing Pg. 32	( )	( )	( )	( )	( )	( )	( )
Protect bird nesting areas using electric fencing Pg. 33	( )	( )	( )	( )	( )	( )	( )
Protect nests with individual exclosures/barriers Pg. 35	( )	( )	( )	( )	( )	( )	( )
Use artificial nests that discourage predation Pg. 45	( )	( )	( )	( )	( )	( )	( )
Use multiple barriers to protect nests Pg. 46	( )	( )	( )	( )	( )	( )	( )
Use snakeskin to deter mammalian nest predators Pg. 47	( )	( )	( )	( )	( )	( )	( )
Use mirrors to deter nest predators Pg. 47	( )	( )	( )	( )	( )	( )	( )
Use lion dung to	( )	( )	( )	( )	( )	( )	( )

deter domestic cats Pg. 50							
Use naphthalene to deter mammalian predators Pg. 48	( )	( )	( )	( )	( )	( )	( )
Use ultrasonic devices to deter cats Pg. 48	( )	( )	( )	( )	( )	( )	( )
Protect nests from ants Pg. 48	( )	( )	( )	( )	( )	( )	( )
Guard nests to prevent predation Pg. 49	( )	( )	( )	( )	( )	( )	( )
Use 'cat curfews' to reduce predation Pg. 50	( )	( )	( )	( )	( )	( )	( )
Play spoken-word radio programmes to deter predators Pg. 50	( )	( )	( )	( )	( )	( )	( )
Plant nesting cover to reduce nest predation Pg. 50	( )	( )	( )	( )	( )	( )	( )
Remove vegetation or perches used by predators, e.g. trees Pg. 53	( )	( )	( )	( )	( )	( )	( )

Reduce mortality by reducing hunting ability or changing predator behaviour

In many circumstances it may be unfeasible or undesirable to control predator numbers or exclude predators from areas. However, it may still be possible to reduce the impact of predators in a variety of ways.

Having read the information on the effectiveness of these interventions provided in the bird synopsis, are you more or less likely to use each intervention in the future?

	**Much more likely**	**More likely**	**No change**	**Less likely**	**Much less likely**	**Don't know**	**NA**
Use collar-mounted devices to reduce predation Pg. 53	( )	( )	( )	( )	( )	( )	( )
Reduce predation by translocating nest boxes Pg. 60	( )	( )	( )	( )	( )	( )	( )
Use supplementary feeding of predators to reduce predation Pg. 54	( )	( )	( )	( )	( )	( )	( )
Use aversive conditioning to reduce nest predation Pg. 55	( )	( )	( )	( )	( )	( )	( )

Final questions and comments

How did you find out about this survey?

We are asking this question to calculate the survey response rate.

( ) A direct email from the researchers

( ) A forwarded email from a colleague

( ) An email through an e-list (please specify which list):
____

( ) Other (please specify): ____

Would you like to receive information about the results and conclusions of this study?

( ) Yes

( ) No

If you have comments about any interventions or general questions or comments about the survey, please feel free use the space below. Alternatively you can contact us personally, either Jessica Walsh (j.walsh@zoo.cam.ac.uk) or William Sutherland (w.sutherland@zoo.cam.ac.uk).

____

____

____

Thank You!

Thank you very much for completing the second part of our survey. We really appreciate your time.

Please have a look at our website www.conservationevidence.com, which provides free summarised information on the effectiveness of a range of management interventions. You will soon be able to access the entire Bird Conservation Synopsis on this website as well.

We wish you all the best in future conservation endeavours.
