# Supplementary material for: The effect of scientific evidence on conservation practitioners’ management decisions
Source: Conserv Biol. 2014 Aug 7;29(1):88–98. doi: 10.1111/cobi.12370 (PMC4515094; doi:10.1111/cobi.12370)
Supplement: Supplementary file 3 [file cobi0029-0088-sd3.pdf]

Appendix S3: Additional Methods

The effect of scientific evidence on conservation practitioners' management decisions

Authors: Jessica C. Walsh*, Lynn V. Dicks and William J. Sutherland
* j.walsh@zoo.cam.ac.uk

Surveys for practitioners

Online surveys were ideal for this study as they are a convenient and efficient method to capture quantitative and qualitative data from large sample sizes across wide spatial distributions (Newing 2011). We followed an online survey distribution protocol, which included an invitation email with the survey link, one or two reminder emails and a thank-you email for the two separate surveys (Dillman et al. 2009). We sent the invitation email to 145 contacts within relevant government agencies, non-government organisations, conservation and ecology institutions or societies and consultancies around the world. We asked practitioners in the invitation email and at the end of the survey to forward it on to their relevant colleagues. As a result, we know the survey was sent to relevant practitioners in the following organizations, societies and networks, though this is not an exclusive list: the IUCN SSC Invasive Species Specialist Group, the English, Scottish, Australian, New Zealand, Canadian and Singaporean federal government agencies responsible for bird conservation and predator control, Pacific Invasives Initiative, BirdLife International partners, the Royal Society for the Protection of Birds, New Zealand Landcare Trust and Ecological Society of Australia.

Expert elicitation process

We implemented the following to increase the accuracy and validity of the Delphi process.

1. Using an online, anonymous method ensured that final results were influenced by the quality of scores and comments made by other experts, rather than by dominant personalities in the group and social judgements, which may occur in other expert elicitation methods such as the nominal group technique (Rowe & Wright 1999; Sutherland 2006).
2. In addition to the bird predation experts, we included a few people on the panel who were not specifically bird conservation experts but were able to understand and critically evaluate the content and quality of scientific studies. These people provided an independent assessment of the evidence in the synopsis, without incorporating past experience or biased judgements. It was important for the expert scores of effectiveness and certainty of evidence to reflect the evidence in the synopsis to be comparable with the survey results from the practitioners.
3. The experts were given instructions and guidelines on how to score the interventions (Appendix S4), because providing training and an explanation before the scoring has been shown to improve the accuracy of the final scores (Burgman et al. 2011; Martin et al. 2012).

4. Averages of scores were not presented in the feedback to the experts to reduce the experts' tendencies to move towards the majority of the scores, rather than the most accurate answer based on the rationales and comments from other experts (Bolger & Wright 2011; Bolger et al. 2011).

The variance between expert scores for interventions' effectiveness and certainty of evidence reduced from the first to third rounds, though this was not significant for most interventions (Appendix S3: Figs 1 & 2). On average across interventions, the median of experts' scores for effectiveness and certainty of evidence changed 2.1 points (min=0.0, max=12.5 points) and 0.9 points (min=0.0, max=3.0 points) respectively between rounds 1 and 3. These changes of median scores between rounds indicate an improvement in the experts' judgments compared to the initial scores, though absolute consensus was not reached for most interventions.

Practitioners' awareness of evidence

We calculated an index of the practitioners' prior knowledge of the existing scientific information (P_{ij}) by taking the difference between their scores of how much evidence they thought existed (E_{ij}; the 1-5 score that we converted to 0-100, where 1=0, 2=25, 3=50, 4=75 and 5=100) and the actual certainty of evidence for each intervention (C_j; the median score from the expert panel) and scaling the differences from -1 to +1 by dividing by 100 (Eqn. 1). An index value of $P_{ij} = 0$ meant that practitioner *i* was accurate in their knowledge of the amount of existing scientific information for intervention *j*. A positive P_{ij} value meant that the practitioner thought there was more information than actually existed and a negative value meant that the practitioner underestimated the amount of existing science about an intervention's effectiveness.

(Eqn. 1) $P_{ij} = (E_{ij} - C_j) / 100$

To amalgamate the prior knowledge indices for each practitioner into a meaningful 'awareness of evidence' score (A_i) we calculated the mean of the absolute values of their prior knowledge of evidence (P_{ij}) across interventions for each practitioner, and then subtracted it from 1 to correct the direction of the scale (Eqn. 2). The value *n* was the number of interventions that were relevant for each practitioner (i.e. excluding interventions they answered as 'not relevant' in the second survey). This awareness score was equal to 1 if a practitioner had perfect knowledge of the extent of research for all interventions and 0 if they were as wrong as possible for all interventions.

(Eqn. 2) $A_i = 1 - (\Sigma |P_{ij}|) / n$

Literature cited

Bolger, F., A. Stranieri, G. Wright, and J. Yearwood. 2011. Does the Delphi process lead to increased accuracy in group-based judgmental forecasts or does it simply induce consensus amongst judgmental forecasters? Technological Forecasting and Social Change **78**:1671–1680.

Bolger, F., and G. Wright. 2011. Improving the Delphi process: lessons from social psychological research. Technological Forecasting and Social Change **78**:1500–1513.

Burgman, M., A. Carr, L. Godden, R. Gregory, M. McBride, L. Flander, and L. Maguire. 2011. Redefining expertise and improving ecological judgment. Conservation Letters **4**:81–87.

Dillman, D. A., J. D. Smyth, and L. M. Christian. 2009. Internet, mail and mixed-mode surveys: the tailored design method. Page 499.

Martin, T. G., M. A. Burgman, F. Fidler, P. M. Kuhnert, S. Low-Choy, M. McBride, and K. Mengersen. 2012. Eliciting expert knowledge in conservation science. Conservation Biology **26**:29–38.

Newing, H. 2011. Conducting research in conservation: social science methods and practice. Pages 1–376. Routledge, London.

Rowe, G., and G. Wright. 1999. The Delphi technique as a forecasting tool: issues and analysis. International Journal of Forecasting **15**:353–375.

Sutherland, W. J. 2006. Predicting the ecological consequences of environmental change: a review of the methods. Journal of Applied Ecology **43**:599–616.

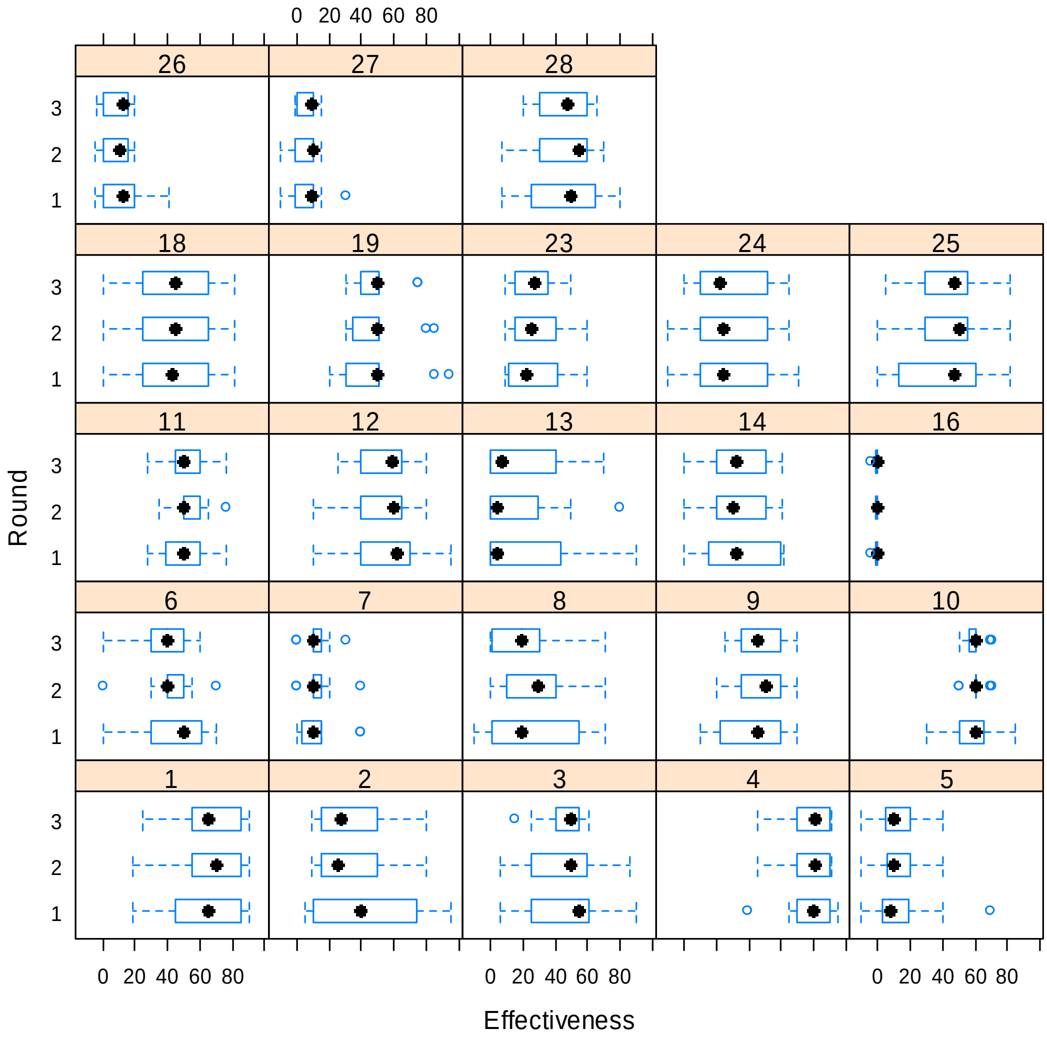

Figure 1: Boxplots of the 10 experts' effectiveness scores for each intervention in rounds 1, 2 and 3 of the Delphi process. Only three interventions had significant reductions in variance for their scores of effectiveness from round 1 to round 3: intervention 3 (p=0.034), intervention 10 (p=0.021) and intervention 27 (p=0.038), tested using one-sided "greater than" F-tests.

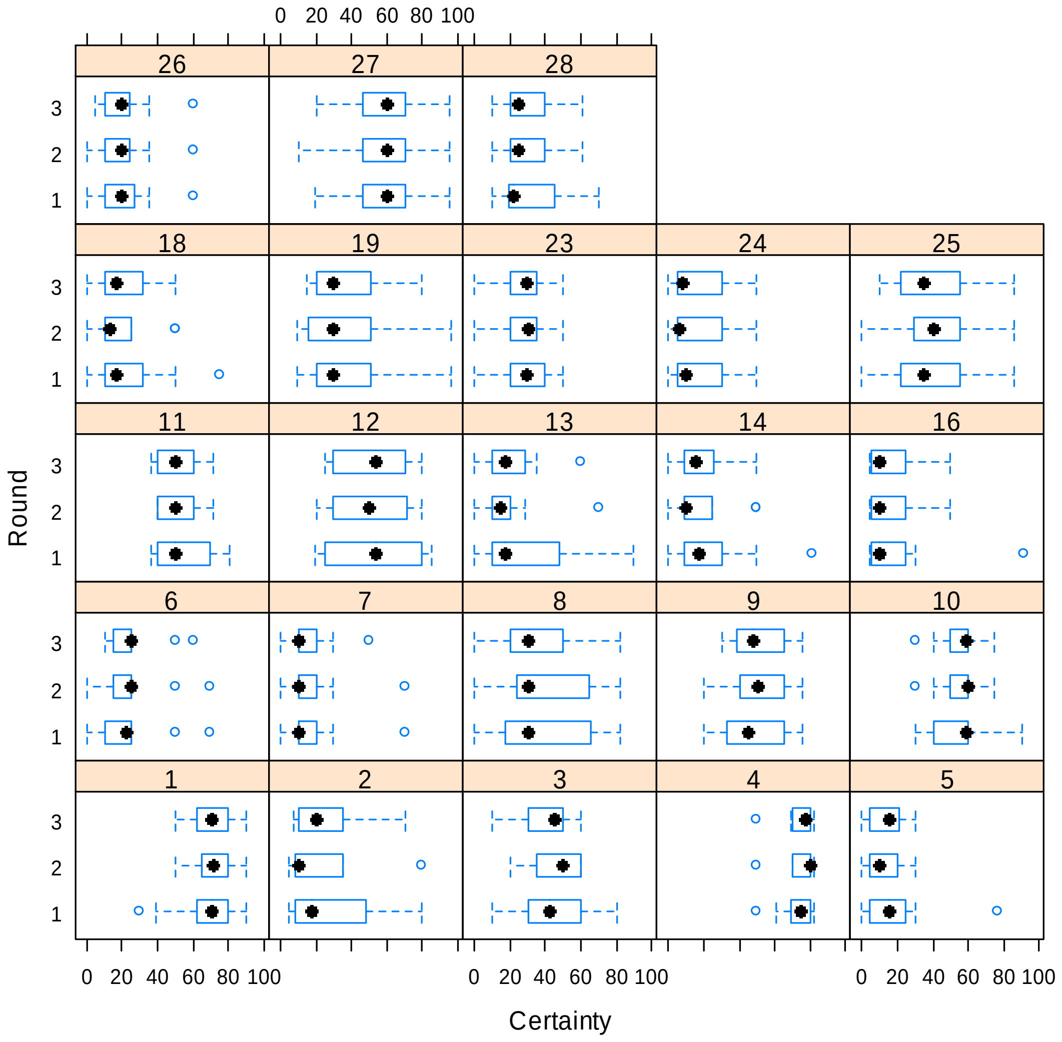

Figure 2: Boxplots of the 10 experts' certainty of evidence scores for each intervention in rounds 1, 2 and 3 of the Delphi process. One intervention had a significant reduction in variance for the scores of certainty of evidence from round 1 to round 3: intervention 5 (p=0.015), tested using one-sided "greater than" F-tests.
