# Supplementary material for: The effect of scientific evidence on conservation practitioners’ management decisions
Source: Conserv Biol. 2014 Aug 7;29(1):88–98. doi: 10.1111/cobi.12370 (PMC4515094; doi:10.1111/cobi.12370)
Supplement: Supplementary file 4 [file cobi0029-0088-sd4.pdf]

The Delphi Process

The Delphi process was developed in the 1960s as a method of capturing expert judgements to forecast future events.

It has been applied in various scientific, political and decision making situations
- when accurate data are not available,
- when expert judgement is the only source of reliable information, and
- when the true answer to a question is unknown.

Why are we using expert judgement and the Delphi process?

We need a panel of experts for this study to interpret the evidence for the effectiveness of management interventions to reduce bird predation. The average of several experts' opinions is likely to be more reliable and accurate than the opinion of a single expert.

Other methods to elicit expert opinion involve participatory discussion before reaching a consensus. This may bias results towards opinions of the dominant, more experienced members of the expert panel.

Instead, in the Delphi process members of the expert panel remain anonymous throughout scoring. Final results are influenced by the quality of scores and comments made by other experts, rather than by dominant personalities and social judgements.

Using an online platform allows people from around the world to participate in the expert panel, engaging with people from a wide range of backgrounds. There is evidence that including a breadth of backgrounds enhances the benefits of the Delphi process.

UNIVERSITY OF CAMBRIDGE
Department of Zoology

What is involved?

The Delphi process improves the judgement of each expert and reduces the variance between the individual scores of the panellists, converging on the 'truth' or the agreed answer.

The Delphi process involves two to three rounds of anonymous scoring. After making an initial judgement, experts are shown a summary of the results and comments from others in the expert panel. They are then asked to reassess their scoring and justify their decisions to change or retain the same scores.

An expert may not have considered several important factors that influenced other experts. Experts should improve their own scoring with this additional information.

The Delphi Process

**Facilitator**		**Experts on panel**
Round 1 – sends instructions, evidence and questionnaire to experts	→	Round 1 – read evidence and make initial scores based on opinion
	↙	
Round 2 – collates Round 1 results and produces summary of scores and comments	→	Round 2 – read summary of results and decide whether to change scores, providing justification
	↙	
Round 3 – collates Round 2 results and produces summary of scores and comments	→	Round 3 – read summary of Round 2 results and decide to change scores or retain past scoring
	↙	
Finish – collates Round 3 results and produces finalised scores		

For more information

- *Technological Forecasting and Social Change* dedicated to the Delphi Process (2011) vol. **78**, issue 9.
- Burgman, M. *et al.* (2011) *Conservation Letters*, **4**: 81-87.
- Martin, T. G. *et al*. (2012) *Conservation Biology*, **26**: 29-38.

Instructions for the Bird Predation Study

Authors: Jessica C. Walsh*, Lynn V. Dicks and William J. Sutherland
* j.walsh@zoo.cam.ac.uk

1. *What do we mean by 'effectiveness' of an intervention?*

This is a difficult concept, as there are a range of scales and interpretations upon which we could measure the effect of an intervention at reducing predation on birds. For example, effectiveness could be measured by the frequency of it working, the magnitude of positive change, the number of specific species or bird groups it targets and its time and spatial scales.

In addition, the type of impact an intervention has on bird species is important to consider in the effectiveness measure. An intervention that has been shown to increase the population of a species or diversity of a bird community is more 'effective' than an intervention that increases hatchling and reproductive success or decreases rates of predation. We also believe that an intervention is more effective if there is a visible impact on the bird species of concern, rather than a decrease in the predator's population, presence or hunting ability.

We want you to take all of these factors into account and score the effectiveness of an intervention, based on the evidence that is provided in the Bird Conservation Synopsis. The 'effectiveness' scale is from -10 to 100. A rating of -10 is for interventions that are harmful, that cause increases in bird predation or have trade-offs with other conservation objectives.

Rating	Meaning
-10	Harmful or trade-offs
0	Ineffective
25	Slightly effective
50	Moderately effective
75	Very effective
100	Always effective
Uncertain	Insufficient evidence

You can slide the pointer along the scale to the rating that you think is most accurate (see example below). If the evidence in the synopsis is insufficient to make a decision and you are unable to rate the effectiveness of an intervention, it is perfectly acceptable to tick the 'Uncertain' box at the end of the slider.

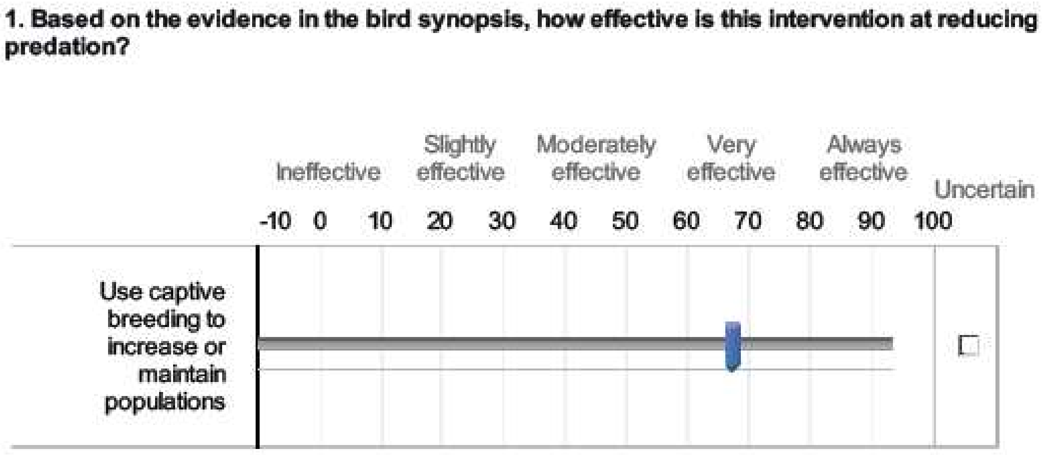

It is important to only consider the evidence that is available in the synopsis. Please try to base your judgement on the published literature, while ignoring your past experience. We understand that this may be difficult and slightly unrealistic in a management scenario. However, it is an essential for the purposes of this research. If an important study is missing from the synopsis, please mention it in the comments section (Question 3, see below).

2. What do we mean by ‘certainty of evidence?

The ‘certainty of evidence’ is quite different to the effectiveness measure. It is a rating of how certain you are about the effectiveness score you gave. It is a combination of the quality and quantify of evidence provided for each intervention and the similarity of results across different studies.

The quality of a study is determined by the experiment design. For example, studies that use randomised, replicated controlled experiments with large sample sizes are of higher quality than studies that use before and after comparisons or single site trials.

We will ask you to rate the certainty of evidence for each intervention on a scale of 0-100%. Try to answer the question by thinking about how much more evidence do we need to be 100% certain of the effect of this intervention.

Rating	Meaning
0%	No useful evidence available
25%	Little quality evidence, low certainty
50%	Some quality evidence, moderate certainty
75%	A lot of quality evidence, high certainty
100%	Fully resolved with high quality evidence

To rate the certainty of evidence for each intervention, move the pointer to the score that you think is most appropriate. If you have comments relating to an intervention, please add them in Question 3 (see below).

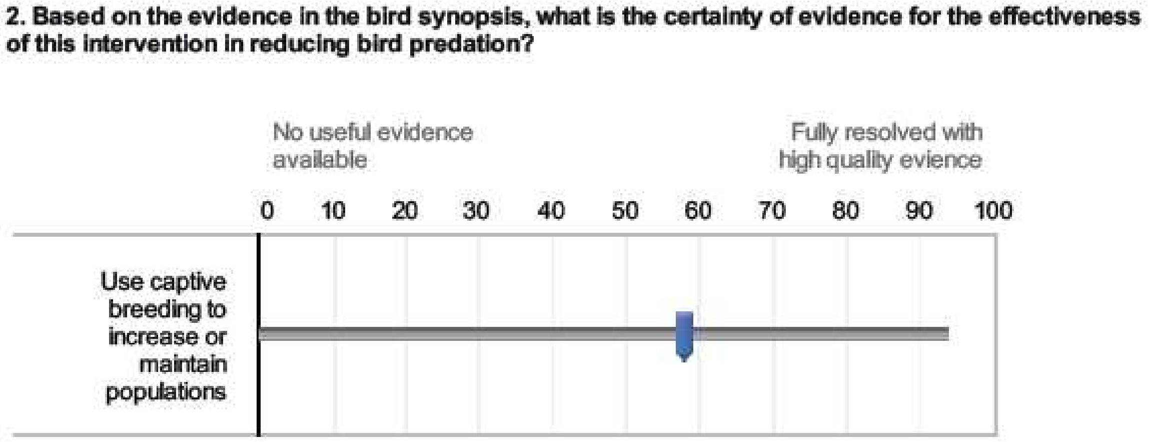

3. Providing useful rational statements and comments

We will then ask you for comments about each intervention. These will form an important component of the summary of results which we give you in the second round of scoring. This is your opportunity to tell others on the expert panel about a relevant piece of information that is not included in the synopsis or to provide a reason for why you scored the way you did. This will be anonymous and your name will not be given out to other experts on the panel.

The comments should be

- stated clearly so that others on the expert panel will understand them
- based on causal arguments, rather than personal opinion or preference
- providing information or other studies that are not captured in the bird synopsis

Comments that do not contribute any valuable insight to the debate will not be included in the summary report. This will ensure that experts are not influenced by uninformative comments and encourage experts to fully engage and provide useful justifications, which will ultimately improve the accuracy of the results collected through the Delphi process.

If you rate an intervention higher or lower than the majority of experts, your rational will be used to defend your position in the second round. On the other hand, if your rating for an intervention is within the majority of scores, your rational should be used to influence the experts on the peripheries to move towards the centre.

An example of a good, well reasoned justification

3. Please provide a comment about captive breeding or a justified reason for your rating on its effectiveness or certainty of evidence.

The evidence shows that most captive breeding attempts have been successful, with only a few examples where birds did not breed in captivity, or when hatchling success was low. The evidence is of high quality because there are multiple studies across different bird groups showing similar results. However, it is important to remember the trade-off impacts of taking birds out of their natural habitat for the breeding programme and the potential problems with re-introduction of captive-bred offspring into the wild.

An example of a poorly justified, subjective comment

3. Please provide a comment about captive breeding or a justified reason for your rating on its effectiveness or certainty of evidence.

I have tried captive breeding and it works well. Even though the method is expensive, I think it is a good way of conserving birds and showing the public that we are doing a good job. I am friends with most of the authors who have conducted these studies and think they work hard for the results they produce.
